# Xylose Fermentation by *Saccharomyces cerevisiae*: Challenges and Prospects

**DOI:** 10.3390/ijms17030207

**Published:** 2016-02-25

**Authors:** Danuza Nogueira Moysés, Viviane Castelo Branco Reis, João Ricardo Moreira de Almeida, Lidia Maria Pepe de Moraes, Fernando Araripe Gonçalves Torres

**Affiliations:** 1 Departamento de Biologia Celular, Universidade de Brasília, Brasília, DF 70910-900, Brazil; danuzamoyses@petrobras.com.br (D.N.M.); vireis@unb.br (V.C.B.R.); lmoares@unb.br (L.M.P.M.); 2 Petrobras Research and Development Center, Biotechnology Management, Rio de Janeiro, RJ 21941-915, Brazil; 3 Embrapa Agroenergia, Laboratório de Genética e Biotecnologia, Parque Estação Biológica s/n, Av. W3 Norte, Brasília, DF 70770-901, Brazil; joao.almeida@embrapa.br

**Keywords:** xylose, lignocellulosic ethanol, fermentation, *Saccharomyces cerevisiae*

## Abstract

Many years have passed since the first genetically modified *Saccharomyces cerevisiae* strains capable of fermenting xylose were obtained with the promise of an environmentally sustainable solution for the conversion of the abundant lignocellulosic biomass to ethanol. Several challenges emerged from these first experiences, most of them related to solving redox imbalances, discovering new pathways for xylose utilization, modulation of the expression of genes of the non-oxidative pentose phosphate pathway, and reduction of xylitol formation. Strategies on evolutionary engineering were used to improve fermentation kinetics, but the resulting strains were still far from industrial application. Lignocellulosic hydrolysates proved to have different inhibitors derived from lignin and sugar degradation, along with significant amounts of acetic acid, intrinsically related with biomass deconstruction. This, associated with pH, temperature, high ethanol, and other stress fluctuations presented on large scale fermentations led the search for yeasts with more robust backgrounds, like industrial strains, as engineering targets. Some promising yeasts were obtained both from studies of stress tolerance genes and adaptation on hydrolysates. Since fermentation times on mixed-substrate hydrolysates were still not cost-effective, the more selective search for new or engineered sugar transporters for xylose are still the focus of many recent studies. These challenges, as well as under-appreciated process strategies, will be discussed in this review.

## 1. Introduction

The rapid depletion of fossil fuel reserves, global warming, and geopolitical issues has triggered a worldwide quest for sustainable renewable energy sources. Several biofuels have been considered but bioethanol remains as the most widely used alternative to conventional fuels for several reasons, including its high octane value and efficient combustion.

Although some bacteria, such as *Zymomonas mobilis* and genetically-modified *Escherichia coli*, are capable of fermenting sugars [1], the yeast *Saccharomyces cerevisiae* is still the organism of choice for industrial production of ethanol. This is essentially due to its high ethanol tolerance and the ability to ferment under strictly anaerobic conditions. Additionally, unlike its prokaryotic counterparts, *S. cerevisiae* withstands low pH and is insensitive to bacteriophage infection, which is particularly relevant in large industrial processes.

Currently, bioethanol is produced either from starch or from the sucrose fraction of some edible agricultural crops, such as corn, sugar cane, and sugar beet. For economic and environmental reasons agricultural residues and other low-value sources of carbohydrates are highly considered for bioethanol production [2]. These include corn stover, sugar cane bagasse, wheat straw, non-recyclable paper, and switchgrass. Lignocellulosic biomass is essentially composed of cellulose, hemicellulose, pectin, and lignin [3], with glucose being the main sugar constituent, but pentose sugars, such as d-xylose and l-arabinose, may represent up to 20% [4]. Despite its immense potential, the use of lignocellulosic substrates for bioethanol production faces three main challenges:

1. A pre-treatment step involving the use of extreme physicochemical conditions and hydrolytic enzymes is required to release fermentable sugars [5,6];
2. Some compounds derived from the pre-treatment steps (e.g., furaldehydes, acetate, formate, phenolic derivatives) are known to inhibit fermentation [7,8];
3. Pentoses are not readily fermented by *S. cerevisiae* [3,9].

Although pentose fermentation is accomplished by non-*Saccharomyces* yeasts, such as *Kluyveromyces marxianus*, *Scheffersomyces* (*Pichia*) *stipitis*, *Pachysolen tannophilus*, and *Candida shehatae*, [10,11] they have not been considered for large-scale processes. Hence, most work involving pentose fermentation in yeast is focused on generating genetically-modified *S. cerevisiae* strains with heterologous xylose metabolic pathways. The challenges are innumerous and will be discussed in this review.

## 2. Xylose Metabolic Pathways

**Figure 1:**
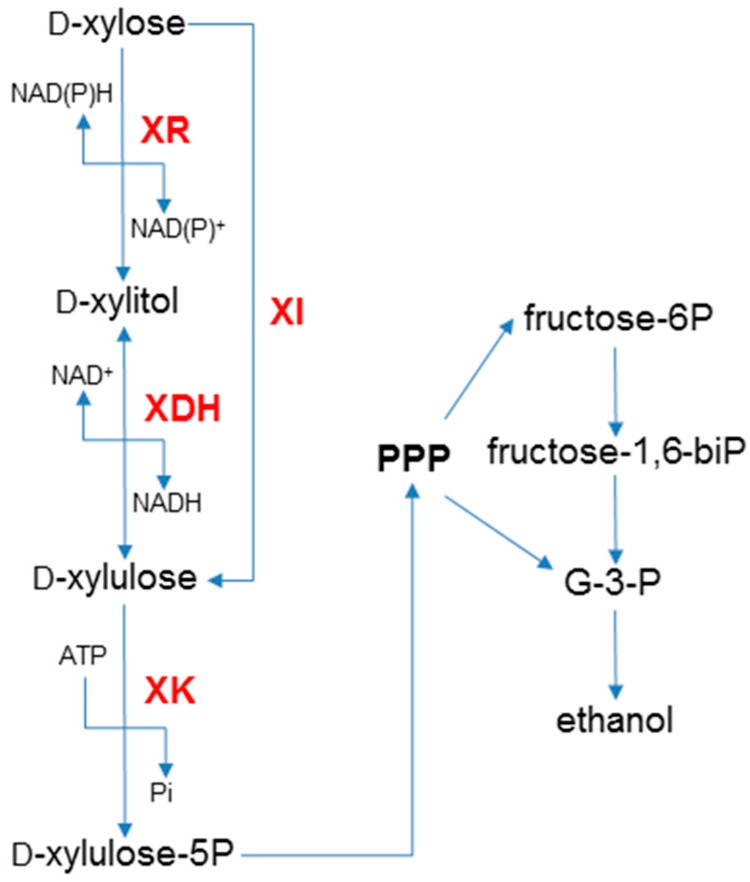
Xylose fermentation in *S. cerevisiae*. The fungal pathway uses xylose reductase (XR) and xylitol dehydrogenase (XDH,) whereas the bacterial pathway uses xylose isomerase (XI). Both pathways produce d-xylulose which is converted to d-xylulose-5P by endogenous xylulokinase (XK). d-Xylulose-5P then enters the pentose phosphate pathway (PPP) where it is further metabolized to form ethanol under appropriate conditions. Arrows indicate the direction of the chemical reactions.

Xylose catabolism occurs through three different pathways in microorganisms, but only two have been introduced into *S. cerevisiae* (Figure 1) [12,13]. Filamentous fungi and some yeasts use an oxidoredutive pathway which involves two reactions. First, xylose is reduced to xylitol by a NAD(P)H-dependent xylose reductase (XR) encoded by *XYL1* [14]. Then, xylitol is oxidized to 5-xylulose by a NADP^+^-dependent xylitol dehydrogenase (XDH) encoded by *XYL2* [15]. Bacteria use a xylose isomerase pathway (XI) to convert xylose directly to 5-xylulose (reviewed in [16]). In both pathways, 5-xylulose is phosphorylated to 5P-xylulose, which is further metabolized through the pentose phosphate pathway (PPP) and glycolysis.

Unlike fungi and bacteria, Archae possess an oxidative xylose catabolic pathway. In this pathway, xylose is oxidized exclusively to the tricarboxylic acid cycle intermediate α-ketoglutarate through upon the action of xylose dehydrogenase, xylonate dehydratase, 2-keto-3-deoxyxylonate dehydratase, and α-ketoglutarate semialdehyde dehydrogenase [17]. This pathway shows similarities to proposed oxidative pentose degradation pathways to α-ketoglutarate found in few bacteria, e.g., *Azospirillum brasilense* and *Caulobacter crescentus*, and in the Archaeon *Sulfolobus solfataricus* [13]. Since this pathway involves many genes it has not been used yet to engineer *S. cerevisiae*. On the other hand, as will be discussed below, both XR/XDH and XI pathways have been extensively employed to construct *S. cerevisiae* strains capable of fermenting xylose with different rates of success.

## 3. Engineering *Saccharomyces* with the XR/XDH Pathway

Despite orthologous genes encoding functional XR and XDH have been identified in *S. cerevisiae*, this yeast is not able to grow solely on xylose [18,19]. Overexpression of native aldose reductase and xylitol dehydrogenase genes allowed growth on xylose, albeit with limited growth rates [20]. Even before this native pathway for xylose consumption was identified, heterologous pathways from naturally xylose-assimilating yeasts were used to engineer *S. cerevisiae*. The *S. stipitis* XR/XDH pathway is the most frequently used to engineer yeast for xylose fermentation, although an important limitation was identified; while XR preferentially uses NADPH as a cofactor, XDH exclusively uses NAD^+^ [16] (Figure 1). This leads to xylitol excretion due to cofactor imbalance, reducing carbon assimilation and ethanol production in the engineered strains. Several strategies have been employed to solve this problem, the redirection of carbon fluxes from NADPH to NADH consuming reactions being the most common denominator. This includes an adding external electron acceptor to the fermentation media [21,22], connecting furaldehyde reduction with xylose metabolism [23], altering the ammonium assimilation pathway [24], channeling carbon fluxes through a recombinant phosphoketolase pathway in a xylose-consuming strain [25], and altering cofactor preference of XR and XDH [26]. These strategies normally result in engineered strains with lower yields of xylitol production.

## 4. Engineering *Saccharomyces* with the XI Pathway

Despite presenting the advantage of not requiring pyridine nucleotide cofactors many prokaryotic XI (encoded by *xylA*) showed no activity when expressed in *S. cerevisiae* [27,28,29,30]. This was attributed to several reasons, including protein misfolding, post-translational modification, improper disulfide bridge formation, sub-optimal internal pH, and absence of specific metal ions [31].

The first functional *xylA* expressed in yeast was that from *Thermus thermophiles* which showed low activity at 30 °C because its maximal activity occurs at 85 °C [32]. A mutant *TtxylA* showed an improvement in ethanol yield at 40 °C, but no production occurred at 30 °C [33]. Since then, other prokaryotic XI were expressed in *S. cerevisiae* with moderate success [31,34,35,36].

The first successful recombinant *S. cerevisiae* carrying a XI pathway was obtained with the expression of the eukaryotic *xylA* from the anaerobic fungus *Piromyces* sp E2 [37]. The recombinant strain showed considerably high XI activity of 1.1 U·mg^−1^, but still low growth rates in xylose under aerobic conditions and no growth in anaerobiosis. Prolonged adaptation in xylose under anaerobic conditions resulted in the isolation of a strain (RWB202-AFX) which showed a specific growth rate of 0.03 h^−1^ and ethanol yield of 0.42 g·g^−1^ [38].

The poor rates of xylose fermentation in *S. cerevisiae* have been attributed to xylitol formation, by the activity of an endogenous non-specific aldose reductase (Gre3) and limited activity of xylulokinase (XK) and non-oxidative PPP enzymes [30]. In an attempt to overcome these metabolic bottlenecks, the genes encoding XK, ribulose 5-phosphate isomerase, ribulose 5-phosphate epimerase, transketolase, and transaldolase were overexpressed in strain RWB202-AFX and *GRE3* was deleted [39]. The resulting strain was submitted to anaerobic adaptation in xylose and a clone (RWB218) was obtained with a specific growth rate of 0.12 h^−1^ and ethanol yield of 0.41 g·g^−1^ [40]. Additionally, in batch fermentation in wheat straw hydrolysate the ethanol yield approached the theoretical value (0.51 g·g^−1^) in 55 h and xylitol production was not observed; in fed-batch fermentation on corn stover hydrolysate, ethanol yield was 0.46 g·g^−1^ after 35 h [30]. Likewise, *xylA* from the fungus *Orpinomyces* was functionally expressed in *S. cerevisiae* showing high activity (1.91 U·mg^−1^), but the resulting strain showed slow growth in xylose [41]. This strain was further modified by overexpressing *XKS1* (which encodes XK) and a heterologous sugar transporter (*PsSUT1*) following evolutionary engineering. When grown in a complex medium supplied with 0,01 M borate at 35 °C the resulting strain showed an increment on the growth rate (0.133 h^−1^), ethanol production (Y_E_ = 0.48 g·g^−1^ and Q_E_ = 0.191 g·L^−1^·h^−1^), and low xylitol production (0.04 g·g^−1^) [41,42].

XI from the anaerobic bacterium *Clostridium phytofermentans* (*CpXylA*) was successfully expressed in an industrial *S. cerevisiae* strain [43]. The main advantage of *CpXylA* is that it shows low susceptibility to xylitol inhibition with a K_i_ three times smaller than that of *PyXylA*. Anaerobic batch fermentation with the recombinant yeast strain expressing *CpXylA* showed ethanol productivity of 0.03 g·L^−1^·h^−1^ and ethanol yield of 0.43 g·g^−1^, with xylitol production of 0.18 g·g^−1^. XI and XK from *Prevotella ruminicola* were co-expressed in *S. cerevisiae*, which was also submitted to evolutionary engineering, resulting in a 3.8-fold increase in specific growth rate (0.23 h^−1^). Microaerobic fermentation using the adapted strain in xylose showed 0.041 g·g^−1^·h^−1^ productivity, 4.8 g·L^−1^ xylitol, and 82.9% of the theoretical ethanol yield [44].

An evolved recombinant yeast expressing *Burkholderia cenocepacia xylA* showed the best ethanol yield (0.45 g·g^−1^) and productivity (0.42 g·cell^−1^·h^−1^) described so far. This strain does not accumulate xylitol and co-consumes glucose and xylose under anaerobic conditions [45,46]. Recently, the activity of four *xylA* genes from mammal gut bacteroidetes (*Bacteroides vulgatus*, *Tannerella* sp., *Paraprevotella xylaniphila*, and *Alistipes* sp.) were assessed in two *S. cerevisiae* strains: one overexpressing XK and non-oxidative PPP enzymes and with a disrupted *GRE3* gene, and another with an additional disruption of *CYC3* [47]. Both strains were evolved in xylose medium with a plasmid expressing XI from *B. vulgatus*. After that, the adapted strain was cured and transformed with plasmids expressing *xylA* from the other different sources described above. All recombinant clones were able to ferment xylose, thus highlighting the usefulness of a xylose-adapted strain to screen for XI activity in *S. cerevisiae*.

It is known that *S. cerevisiae* naturally metabolizes xylulose [48]. This observation led to an interesting approach for ethanol production which is based on the secretion of XK. The cell surface display of *XylA* from *Clostridium cellulovorans*, together with overexpression of *ScXKS1*, led the recombinant yeast to produced 0.5 g·L^−1^ ethanol under anaerobic conditions [49].

## 5. Additional Genetic Modifications

From the results shown above it is clear that the simple introduction of the XR/XDH or XI pathways is not enough to ensure effective xylose fermentation by the recombinant yeast. Kinetic modeling shows that a higher level of XK expression and overexpression of sugar transporters (which will be discussed later) are the most advantageous modifications in recombinant yeast [50]. However, overexpression of XK may affect yeast growth and ethanol production [51]. Other important enzymes for xylose metabolism are non-oxidative PPP enzymes with transaldolase playing a key role as it limits xylose flux [52].

Other important genetic modifications involve the elimination of competitive pathways and the increase of cofactor regeneration in oxidoreduction pathway. Competitive pathways can convert intracellular xylose to xylitol, thus leading to xylitol accumulation. In the case of the XI pathway, xylitol accumulation is undesirable not only by draining intracellular xylose, but also it inhibits most XI and breaks metabolic flux [53]. In *S. cerevisiae* the deletion of *GRE3*, an unspecific aldose reductase that can convert xylose to xylitol [54], is an important step to decrease xylitol production [55]. In the case of the oxidoreduction pathway, the overexpression of *GDP1* (a fungal NADP^+^-dependent d-glyceraldehyde-3-phosphate dehydrogenase from *Kluyveromyces lactis*) regenerates NADPH without CO_2_ production, thus facilitating anaerobic xylose fermentation. Additionally, deletion of *ZWF1* (glucose-6-phosphate dehydrogenase), which catalyzes the first step of the pentose phosphate pathway, avoids competition for NADPH production [56]. Additionally, deletion of *PHO13* (a phosphatase) in recombinant yeast overexpressing *S. stipitis XYL3* (XK), increased yeast growth and ethanol production in a xylose medium [57].

*In silico* models are an important tool to analyze an engineered microorganism for the efficient production of a desired product from new substrates. One such model proposes that in recombinant yeast for the oxidoreduction pathway, the deletion of the NADP^+^-dependent glutamate dehydrogenase gene (*GDH1*) and overexpression of the NAD^+^-dependent *GDH2* increases the specificity of XR for NADH [23]. This strategy led to decreased xylitol byproduct and increased ethanol yield. Other examples show that when *GAPN* (non-phosphorylating NADP^+^-dependent glyceraldehydes-3-phosphate dehydrogenase) is expressed in xylose-recombinant yeast, ethanol yield increases up to 25% in a glucose/xylose mixture with reduction in glycerol and xylitol formation [58].

Furthermore, these genetic modifications more directly linked to xylose consumption pathway, and there are some others genes detected through synthetic genomic array (SGA) whose modification affected xylose consumption. SGA identified four genes (*ALP1*, arginine transporter; *ISC1*, mitochondrial membrane-localized inositol phosphosphingolipid phospholipase; *RPL20B*, component of 60S larger ribosomal subunit; *BUD21*, a component of small ribosomal subunit) that, when individually deleted, improved xylose consumption to levels similar to those of recombinant XI/XK yeasts [59]. Although it is not clear how these genes act on xylose metabolism this result shows that adjustments on these unpredicted targets are important in order to obtain improvements in xylose consumption.

## 6. Evolutionary Engineering

As mentioned previously, the introduction of a xylose pathway in *S. cerevisiae* is not enough to promote efficient conversion of xylose to ethanol since it is often the case that a desired phenotype is not associated to a single gene or to any obvious modification [60]. Pathway networks often interact with each other, as well as with the exogenous pathway, promoting many challenges for the production of the desired product [61]. Additionally, overexpression or gene knockout is not sufficient to obtain the expected outcome, as it is often needed to perform extensive transcription re-programming according to metabolic flux.

One method which permits the selection of simultaneous genetic modifications in network pathways in order to improve its performance is evolutionary engineering, involving reprogramming the cell’s innate metabolism at different levels. This method is based on adaptive evolution, a natural process in which organisms undergo mutations that confer a tolerance to environmental changes [62]. Natural evolution is slow, but there are means to accelerate this process, mainly for microorganisms because of their fast growth. The microorganism is placed under environmental selection and after some time the survived population will exhibit the desired phenotype due to “natural” mutations which permitted adaptation. Another approach involves yeast mutagenesis (chemical or physical), followed by selection of the desired phenotype. For example, when *S. cerevisiae* with no genetic modifications for xylose consumption was evolved in xylose medium, the resulting strain had the activities of endogenous XR and XDH increased seven- and 60-fold, respectively [63].

Combining mutagenesis and evolutionary engineering approaches is often the best choice to obtain the desired phenotype. For example, after recombinant TMB3001 yeast strain expressing *XYL1*, *XYL2I*, and *XKS1* was submitted to EMS mutagenesis, followed by three rounds of metabolic adaptation, some isolated clones had improved ethanol production and others only consumed xylose [64]. Other works involving adaptive evolution of the oxidoreduction pathway report improved ethanol production [65,66,67].

A recombinant industrial strain for *CpXylA* was submitted to EMS mutagenesis, genome shuffling, and evolutionary engineering in a complex medium with d-xylose [68]. The resulting strain produced 32% more ethanol than the parental strain and XI activity had a 17-fold improvement. In another study, a recombinant strain for *PyXylA* was obtained with overexpression of PPP enzymes and XK and was further submitted to evolutionary engineering. The final strain showed one of the best ethanol productions so far (Y_E_ = 0.438 g·g^−1^, Q = 0.687 g·g^−1^·h^−1^), with a specific growth rate of 0.148 h^−1^ and very low xylitol production (Y_X_ < 0.01 g·g^−1^) [69]. Inverse metabolic engineering identified 32 copies of *PyXylA* in the final evolved strain and global transcriptional analysis showed that the xylose pathway and PPP had overexpressed genes. Additionally, the fermentative pathway and amino acids metabolism genes showed altered levels of expression in the evolved strain.

The amplification of gene-encoding XI seems to be an important consequence of yeast evolutionary adaption on xylose. Recently, it was shown that the adaptation on xylose of an industrial yeast strain carrying integrated *CpXylA* involved the formation of an extrachromosomal circular DNA element (eccDNA) intermediate [70]. This intermediate, which carried *CpXylA* and an ARS (autonomous replicating sequence) element, eventually integrated in tandem at the original locus, resulting in a strain with high xylose fermentation capacity.

A powerful genetic tool called global transcription machinery engineering (gTME), which allows reprogramming the innate metabolism of the cell by creating mutations in key proteins involved in transcription, has been successfully used to perform global transcription changes. For example, an improvement in ethanol and glucose tolerance in yeast was obtained by using a *spt15* mutant library [71]. *SPT15* is a TATA-binding protein which has an important role in promoter specificity. Additionally, new genes involved in ethanol tolerance were identified by using gTME [72]. Despite its usefulness, few works have described the use of this technique for xylose consumption. When gTME was applied in *S. cerevisiae* for xylose fermentation, the final strain had improved the capacity to consume xylose, but ethanol production had decreased [73]. In another work, gTME was used to adapt *S. cerevisiae* to corn cob hydrolysate as a substrate [74]; the resulting strain produced 11.9 g·L^−1^ ethanol after 71 h.

## 7. Xylose Uptake

Many *S. cerevisiae* strains have been genetically engineered for the conversion of xylose into ethanol by improving metabolic flux for xylose utilization [75]. Most of them rely on the capacity of endogenous hexose transporters for xylose uptake, since *S. cerevisiae* lacks a xylose-specific transport system. Studies using evolutionary engineering strategies to obtain fast xylose-utilizing yeast resulted in increased expression of hexose transporters in the evolved strain [40,46,76]. Overexpression of both heterologous and native transporters may improve ethanol productivity [77,78,79,80]. However, the lack of efficient xylose transporters is only evident when xylose metabolism is not rate-limiting or when xylose concentration decreases down to 0.05%–0.4%, due to the low affinity of nonspecific transporters [75,78].

*S. cerevisiae* has 18 hexose transporters (Hxt1-17 and Gal2) capable of transporting glucose into the cell, although only seven (Hxt1-7) are responsible for glucose internalization acting as facilitators. They are divided into three types of glucose transporters according to their affinities for glucose: high-affinity transporters Hxt6, Hxt7, and Gal2 (with *K*_m_ 1–2 mM); intermediate-affinity transporters Hxt2, Hxt4, and Hxt5 (with *K*_m_ ~10 mM); and low-affinity transporters Hxt1 and Hxt3 (with *K*_m_ 50–100 mM) [81,82]. The Hxt7, Hxt5, Gal2, Hxt1, and Hxt4 transporters are xylose-permissive, taking up xylose with 200-fold lower affinity and slower rates, depending on the concentration of xylose and glucose outside the cell [83,84]. Hxt7 and Hxt5 seem to be the main transporters responsible for xylose uptake when xylose is the only carbon source available or in co-fermentation when glucose reaches low levels [84]. High concentrations of glucose (>200 mM) severely represses *HXT7* and *HXT5* transcription, while inducing transcription of *HXT1* and *HXT3*, but when glucose concentration drops below 2% in the mixed sugar medium, *HXT4* transcription is induced and, as fermentation proceeds and xylose progressively becomes more available than glucose, *HXT5* is de-repressed and *HXT7* transcription is induced (by low levels of glucose) [83]. *HXT5* and *HXT7* are also expressed in the absence of glucose when *HXT4* and *HXT1* transcription decrease. *HXT1* showed the highest level of expression when glucose was fermented, while *HXT7* had the highest level when xylose was fermented [80,83]. It was shown that a low concentration of glucose (0.5 g·L^−1^) could improve xylose utilization, when compared with xylose alone [85]. This should be due to the inducible increase in the expression of *HXT7* by low glucose levels. *GAL2* transcription is repressed in the presence of glucose, but when constitutively expressed, Gal2 and Hxt7 exhibited the highest xylose uptake rates between the endogenous hexose transporters [84] (Table 1).

Glucose and xylose should be co-consumed to reduce fermentation time and to reach productivity compatible to industrial economically-viable processes. However, xylose is usually consumed only after significant glucose depletion from the medium, therefore displaying diauxic fermentation [66]. Simultaneous utilization of xylose and glucose was shown to be limited due to inhibition of xylose uptake by glucose. Since the endogenous hexose transport system favors glucose even when glucose metabolism was disrupted, the inhibitory effect of glucose over the use of xylose remains [86]. To overcome this, several putative genes or known heterologous xylose transporters and engineered endogenous transporters were expressed in *S. cerevisiae* in the last decade [87,88,89,90,91,92] (Table 1). The development of hexose transporter-null strains, such as EBY.VW4000 in which more than 20 hexose transporter genes were deleted, was an important tool for screening and characterization of new transporters by functional complementation of growth on glucose [93], or xylose when a xylose pathway is introduced [84,87,89,91,94]. A strain was used for transporter screening and evolution based on a hexose transporter-null strain with an introduced xylose pathway and disrupted glucose metabolism [95]. The resulting strain was unable to grow on glucose alone, even when a functional hexose transporter was expressed. However, it could grow on mixed sugars if the transporter favored the uptake of xylose over glucose. This was a useful tool for developing transporters with new properties, like enhanced affinity for xylose with no inhibition by glucose.

Unlike *S. cerevisiae*, there are many natural pentose-assimilating species having both low-affinity and high-affinity sugar transport systems for xylose uptake, comprising proton symporters and facilitated-diffusion transporters. Numerous putative genes emerging from increasing genome data seems to be good candidates for new xylose-specific heterologous transporters. More than 80 heterologous transporters have already been expressed in *S. cerevisiae*. *SUT1*, *SUT2*, *XUT1*, *XUT3* (Xyp33), *XUT4*, Xyp29 (*STL12*), *SUT3* (Xyp37) from *S. stipitis* [89,90,91,96,97], *GXS1* and *GXF1* from *Candida intermedia* [87,90], At5g59250 from *Arabidopsis thaliana* [77], An29-2 and An25 from *Neurospora crassa* [89], and *xtrD* from *Aspergillus nidulans* [92], MgT05196 from *Meyerozyma guilliermondii* [98] and *Xylh* from *Debaryomyces hansenii* [99] have been shown to enable the HXT-null *S. cerevisiae* strains to transport xylose. More than 80 percent of these surveyed genes annotated as putative or known sugar transporters were not functional in *S. cerevisiae*, probably due to misfolding or improper localization. While most of them showed preference for glucose over xylose, just three of them were characterized as xylose-specific transporters: An25 and Xyp29 are xylose facilitators, with no glucose uptake activity [89], and recently-reported Xut1, also transport glucose, but with lower affinity and lower *V*_max_ when compared with xylose uptake kinetic parameters (Table 1) [97]. Gxs1, a glucose/xylose H+ symporter, was the first proton symporter from yeast reported that could transport xylose against the concentration gradient [87]. As expected for this type of transporter, it had a high affinity for xylose compared with other transporters (Table 1), but the uptake activity was very slow and the affinity for glucose was higher than for xylose. A conserved motif (G-G/F-XXXG) was identified based on the sequence similarity of *GXS1* with other xylose transporter genes and were able to improve the *V*_max_ two-fold by modifying residues F^38^I^39^M^40^ [91]. This approach was responsible for changing selectivity of the transporter which no longer transported glucose. This result could be used to rewire the native hexose transporter Hxt7 and Rgt2 from *S. stipitis* into xylose-specific transporters (by modifying I^39^M^40^ M^340^ and F^38^M^40^, respectively). However, in all these cases, the uptake activities of these heterologous transporters were too low to dramatically improve co-fermentation productivity [78,89,91,99].

**Table 1:** Kinetic parameters of yeast glucose/xylose transporters.

Transporter	Strain/Plasmid Construction	Strain Characteristics	Xylose		Glucose		Reference
			*K*_m_ (mM)	*V*_max_ (nmol/min/mg)	*K*_m_ (mM)	*V*_max_ (mmol/min/mg)	
ScHxt1	H2219/pYX212-HXT1	Δ*hxt1-7*Δ*gal2*	880 ± 8	750 ± 94	NR	NR	[88]
ScHxt1	KY73/HXT1mnX-pVT	Δ*hxt1-7*Δ*gal2*	NR	NR	46	NR	[100]
ScHxt2	H2219/pYX212-hxt2	Δ*hxt1-7*Δ*gal2*	260 ± 130	340 ± 10	NR	NR	[88]
ScHxt2	KY73/HXT2mnX-pVT	Δ*hxt1-7*Δ*gal2*	NR	NR	3.3	NR	[100]
ScHxt4	H2219/pYX212-HXT4	Δ*hxt1-7*Δ*gal2*	170 ± 120	190 ± 23	NR	NR	[88]
ScHxt7	H2219/pYX212-HXT7		130 ± 10	110 ± 7	NR	NR	
ScHxt7	EBY.VW4000/pRS62N-HXT7	Δ*hxt1-17* Δ*gal2* Δ*stl1* Δ*agt1* Δ*mph2* Δ*mph3* Δ*ydl247w* Δ*yjr160c*	200.3 ± 13.2	67 ± 2	0.5 ± 0.1	26 ± 1.1	[94]
ScHxt7 (N370S)	EBY.VW4000/pRS62N-HXT7-N370S		169.9 ± 26.3	24.1 ±1.6	10.8 ±1.0	47.3 ± 1.2	
ScHxt7	KY73/HXT7mnX-pVT	Δ*hxt1-7*Δ*gal2*	NR	NR	0.67 ± 0.05	610 ± 60 *	[100]
ScHxt7 (T213V)			NR	NR	0.40 ± 0.02	120 *	[101]
ScHxt7 (D340C)			NR	NR	0.43 ± 0.05	430 ± 40 *	[100]
ScGal2	EBY.VW4000/pRS62N-GAL2	Δ*hxt1-17* Δ*gal2* Δ*stl1* Δ*agt1* Δ*mph2* Δ*mph3* Δ*ydl247w* Δ*yjr160c*	225.6 ± 15.8	91.3 ± 3.2	1.5 ± 0.2	27.2 ± 0.9	[94]
ScGal2 (N376F)	EBY.VW4000/pRS62N-GAL2-N376F		91.4 ± 8.9	37.3 ± 1.3	ND	BD	[91]
CiGxf1	TBM3201/pHxt7-GXF1	Δ*hxt1-17* Δ*gal2* Δ*stl1* Δ*agt1* Δ*mph2* Δ*mph3*	48.6 ± 6.5	64.19	2.0 ± 0.6	10.5	[87]
CiGxs1	TBM3201/pHXT7-GXS1		0.4 ± 0.1	6.5 ± 1.5	0.012 ± 0.004	4.3 ± 0.3	
CiGsx1	EBY.VW4000/p414-TEF-CiGXS1	Δ*hxt1-17* Δ*gal2* Δ*stl1* Δ*agt1* Δ*mph2* Δ*mph3* Δ*ydl247w* Δ*yjr160c*	0.026 ± 0.066	0.0072	NR	NR	[91]
CiGsx1 F^38^I^39^M^40^			0.721 ± 0.116	0.015	NR	NR	
SsSut1	RE700/YEpSUT1	Δ*hxt1-7*	145 ± 1.0	132 ± 1.0	1.5 ± 0.1	45.0 ± 1.0	[96]
SsSut4	EBY.VW4000/pRS316-TDHp-SUT1	Δ*hxt1-17* Δ*gal2* Δ*stl1* Δ*agt1* Δ*mph2* Δ*mph3* Δ*ydl247w* Δ*yjr160c*	16.6 ± 0.3	122 ± 2.4	1.3 ± 0.1	105 ± 4.2	[99]
SsXut1	EBY.VW4000/pRS316-TDHp-XUT1		0.46 ± 0.02	116 ± 5.8	0.91 ± 0.01	80 ± 1.0	
SsXyp29	EBY.VW4000/pRS429-HXT7p-XYP29		56 ± 9.4	0.69 ± 0.04	BD	BD	[89]
NcAn25	EBY.VW4000/pRS429-HXT7p-AN25		175.7 ± 21.4	0.61 ± 0.05	BD	BD	
DhXylh	W303-1A/pRS42K-XYLH		1.4 ^†^	1.6 ^†^	NR	NR	[99]
ScHxt36	DS715054/pRS313-HXT7p-HXT36		108	62.5	6	60	[102]
ScHxt36 (N367I)		Δ*glk1* Δ*hxk1* Δ*hxk2* Δ*gal1*	40	23	ND	ND	
ScHxt36 (N367A)			25	29	171	71	

*V*_max_ = mmol/min/mg DW (dry weight); NR, not reported; ND, not determinable; BD, below detectable limit; ^†^ *K*_m_ and *V*_max_ were calculated dismissing the influence of endogenous transport system; * *V*_max_ = рmol/10^7^ cells/5 s; Sc = *Saccharomyces cerevisiae*; Ci = *Candida intermedia*; Ss = *Scheffersomyces stipitis*; Nc = *Neurospora crassa*; Dh = *Debaryomyces hansenii*.

As summarized on Table 1, the native hexose transporters showed the highest xylose uptake activities, even when compared with xylose-specific transporters, like CiGsx1 (F^38^I^39^M^40^), SsXyp29 NcAn25, and SsXut1. Actually, when *HXT7* was constitutively overexpressed in an HXT-null xylose-consuming strain, it could ferment xylose at similar rates as glucose when each sugar was the only carbon source. However, as expected, in mixed sugar medium xylose fermentation was delayed [80]. For Hxt7, substrate affinity (1/*K*_m_ value) is more than 200-fold higher for glucose than for xylose, making this sugar a second choice sugar when competing with glucose, even when glucose is in low concentration. Hence, metabolic engineering strategies to reduce endogenous hexose transporter affinity for glucose or to raise the affinity for xylose—in a way that xylose becomes the first choice sugar in co-fermentation—would probably bring the productivity of ethanol to similar rates as glucose fermentation in a non-xylose-metabolic-rate-limiting strain. Under this scenario, the use of structural characterization data available for Hxt7 and Gal2, with determination of crucial residues involved in glucose affinity, selectivity, and translocation of substrate [100,101], together with the strain developed for evolution and screening of transporters described above [95], boosted studies of evolution of native transporters. A strain with evolved Hxt7 and Gal2 transporters that were no longer inhibited by glucose, after mutating a single residue, Hxt7 (N370S) and Gal2 (N376F) were obtained [94] (Table 1). This substitution led to a decrease in *V*_max_ for both transporters, and completely abolished glucose uptake from Gal2. All of the mutated transporters uninhibited by glucose had a mutation at either N370/376 or T213/219 residues already pointed out elsewhere [101]. Using a similar strategy for transporter evolution, a chimeric Hxt36, a functional glucose transporter constructed by Hxt3-Hxt6 fusion, was evolved [102]. That study used a more severe selection pressure (growth on 0.5%–1% xylose with increasing glucose up to 10%) to obtain an Hxt36 (N367I) transporter, which did not allow growth on glucose (slow growth on xylose) and, by rational amino acid substitution, an Hxt36 (N367A) transporter with lower affinity for glucose than xylose. A strain harboring Hxt36 (N367A) was able to co-ferment glucose and xylose, although consumption rates were lower than for glucose alone, probably due to the reduction in *V*_max_ for xylose (Table 1) [102]. The position N367 of Hxt36 corresponds to the same N370/376 seen before for Hxt7 and Gal2 [94]. As hexose transporter sequences are conserved, these studies of structural homology could be applied to predict rational designs for rewiring new xylose transporters. In conclusion, the strategy of transporter evolution seems to be a promising way to obtain an efficient xylose transporter with high and moderate affinities, such as those encountered in glucose transporters.

## 8. Effects of Lignocellulosic Hydrolysate Inhibitors on Xylose Fermentation

Monosaccharides from cellulose and hemicellulose fibers of biomass must be released before yeast fermentation. For this, a pre-treatment step, which involves the use of extreme physicochemical conditions and an enzymatic hydrolysis step, is applied. In addition to monomeric sugars, several other compounds are released and formed during liquefaction of biomass in the pre-treatment and hydrolysis steps. Indeed, lignocellulosic hydrolysates contain several other compounds and several of them inhibit yeast metabolism. The common groups are organic acids (most usual is acetic acid, derived from hemicellulose deacetylation); furaldehydes (furfural and 5-hydroxymethyl-furfural, formed by dehydration of pentoses and hexoses, respectively); and phenolic derivatives (generated from lignin breakdown) (reviewed in [103]). The presence and concentration of these compounds will vary according to the biomass used, pre-treatment, and hydrolysis conditions [103]. However, even in lower concentrations, these compounds can inhibit yeast metabolism, prolonging the lag phase, damaging cellular membranes, and leading to cytoplasm acidification, thus reducing ethanol yield and productivity [6].

Yeast tolerance to lignocellulosic derived inhibitors is not only strain-dependent, but also dependent of the carbon source to be fermented, once yeast is much more affected when fermenting xylose than glucose [104,105,106,107]. Yeast shows much slower sugar consumption rate for xylose and this may lead to depletion of key metabolites and cofactors that might be especially important in stressful conditions imposed by lignocellulosic hydrolysates [108]. Thus, a number of strategies to improve xylose fermentation performance and concurrently reduce the influence of lignocellulosic hydrolysate inhibitors on recombinant strains have been developed, including construction of recombinant strains expressing detoxifying enzymes [23], transcription factors [106], mutagenesis and breeding [105,109], and evolutionary engineering strategies to increase tolerance to single inhibitors or to the hydrolysate [105,110]. In addition, strategies to improve yeast tolerance by propagation on hydrolysate media before hydrolysate fermentations have been shown to improve yeast performance [111,112]. Despite the success of some strategies, few of the improvements towards lignocellulosic hydrolysate inhibitors were performed in industrial strains. Thus, further studies are needed to elucidate the interactions of xylose metabolism with inhibitor tolerance, and are especially important to generate new, robust industrial strains.

## 9. Insights on Industrial Application

Certainly, there will be no single solution for the development of a yeast (or yeast strains) suitable to all xylose industrial fermentation processes since the challenges discussed above are more or less critical depending on process configurations. For example, xylose uptake is still a challenge only if xylose is co-fermented with glucose; however, adaptation or genetic modification to improve tolerance to inhibitors presented in the hydrolysate would be a greater challenge. The hydrolysate contains inhibitors, such as furaldehydes, phenolic compounds, and weak acids, e.g. acetic acid. This acid is intrinsically related with biomass deconstruction, the advances in pre-treatment process technologies could avoid high levels of the first two inhibitors; however, acetic acid concentration would increase with the improved biomass deconstruction [26]. Under large scale fermentations conditions, there are still more desirable adaptations to tolerate (pH, temperature, high ethanol, and other stress fluctuations), which lead the search for yeasts with more robust backgrounds, like industrial strains, as engineering targets. As presented above, a good fermentation productivity on hydrolysate was achieved by using a genetically-modified industrial strain submitted to evolutionary engineering on hydrolysate, and, additionally, genes that are related to stress tolerance were identified [68]. Recently, the influence of addition of hydrolysate during yeast propagation using an industrial xylose consuming *S. cerevisiae* was shown [112]. This strategy had a huge effect on yeast performance, increasing the final ethanol production yields by 80%.

Although there are plenty of studies on the development of new strains for xylose fermentation, few works have focused on process strategies using these yeasts. A process strategy largely used in first generation ethanol production from sugar cane for cost reduction is cell recycling. A study using immobilized recombinant yeast cells for fermentation of corn stover hemicellulosic hydrolysate showed the possibility of recycling cells for five batches with no significant reduction in ethanol yield [113]. In a process with cell recycling, it would be expected that the diauxic fermentation of mixed sugars could be, to some extent, desirable to control the replacement of a developed and adapted xylose-consuming strain from some derived population less efficient for xylose fermentation. This could lead to the selection of a more robust xylose fermenting population.

## 10. Conclusions

With the global interest in sustainable development with the use of lignocellulosic residues to produce biofuels and other value-added products in the context of biorefineries, it is of utmost importance to improve the ability of *S. cerevisiae* to metabolize xylose. There are still many metabolic bottlenecks that need to be addressed, such as xylose uptake, improvement of catalytic efficiency of xylose isomerases, xylitol formation, and increased specific growth in xylose. In addition to genetic modification by the introduction/modification of native or exogenous metabolic pathways, it is clear that adaptive engineering plays a crucial role in this process. Due to the metabolic complexity involved in the development of xylose-fermenting yeast it is important to use metabolic engineering associated with other modern techniques, such as gTME to accelerate physiological adaptation in xylose. The careful optimization of the engineered yeast by the use of a combination of different approaches should represent a forthcoming challenge. Finally, studies on process strategies with a large variety of newly-developed xylose-fermenting strains should be encouraged in order to establish an economically viable industrial process for ethanol production from xylose-containing lignocellulosic hydrolysates.
